# Lifetime Experience of Violence in Early Adulthood Female

**Published:** 2018-06-30

**Authors:** Kalpana Silwal, Prativa Dhakal, Krishna Bahadur Raut, Rajendra Kumar Chaudhary

**Affiliations:** 1 Chitwan Medical College, School of Nursing, Chitwan, Nepal; 2 Department of Emergency, Chitwan Medical College, Chitwan, Nepal; 3 Department of Obstetrics and Gyanecology, Pokhara Academy of Health Sciences, Kaski, Nepal

**Keywords:** *female*, *prevalence*, *prevention*, *violence*

## Abstract

**Introduction:**

Despite political commitment and a supportive legal and policy framework, violence against women remains a significant problem in Nepal. Nepal Demographic and Health Survey reported more than one in five women experience violence in lifetime. Three fourth of women who had experienced physical or sexual violence had not sought any help. The aim of the study is to find out the status of early adult hood experience of violence in female.

**Methods:**

Descriptive cross-sectional study was carried out in an Institute in Lalitpur. Accessibility sampling was used to find out the experience of violence from their childhood to this date. The data were collected by self-administered questionnaire was distributed to the female students. Data were analyzed using SPSS. Frequency, mean, percentage and standard deviation were calculated.

**Results:**

More than three fourth 71 (79.8%) of the female students were victim of violence and among them most 67 (75.3%) were at age of 11 to 19 years. Majority 63 (70.8%) were victimized from strangers followed by friends 11 (12.4%). Teasing 55 (61.8%) and unwanted touching 35 (39.3%) were the most common type of violence. Most 51 (57.3%) were the victim while traveling by public vehicle and walking on road 47 (52.8%). More than half (57.7%) were suffered <5 times. One third 34 (38.2%) told strict punishment to the offenders followed by awareness program 32 (36%) for the prevention of violence.

**Conclusions:**

Majority of the female students were victim of violence and offenders were young adult. Awareness program, strict rules and punishment to offenders should be implemented to prevent the violence among girls.

## INTRODUCTION

Violence as a result of gender conflicts that pervades social stratification and ethnic groups is reiterated.^1^ College women are at greater risk for rape and sexual assault than women in the general population or in a comparable age group.^2^

The perpetrator in sexual violence crimes knew the victim in 67% of the cases and was a stranger in 33% and seventy-five perpetrators (28%) were members of the victims' families. In 14% of cases, the perpetrator was the victim's father and in 9% her stepfather. The abuse had occurred on multiple occasions in 29 % of the cases.^3^ The occurrence rate of abuse was highest in the summer season (54%).^4^

About 90% of survivors on a college campus know the person who assaulted them.^5^ Case histories revealed that the inability of young women to communicate effectively with their peers and sex partners, lack of self-esteem, job insecurity, and other socioeconomic problems made them vulnerable to these abuses.^6^

The aim of the study is to find out the status of early adult hood experience of violence in female.

## METHODS

A descriptive cross-sectional study was carried out in an Institute in Lalitpur, Nepal from July to August, 2016. Permission was taken from the institute and informed consent was taken from the respondents. Accessibility sampling was used to find out the experience of violence from their childhood to this date. The data were collected by self-administered questionnaire was distributed to the female students. Pre-testing was done among 10% of same level of female students of in another institute in Maharajgunj, Kathmandu, Nepal. Data were edited, coded, tabulated and analyzed by using according to SPSS. The results were interpreted by frequency, percentage, mean and standard deviation.

## RESULTS

Almost half 43 (48.3%) of the students were of age 17 followed by 18 years 26 (29.2%). Majority 73 (82%) of the students followed Hindu religion. Majority 72 (80.9%) of the students were from nuclear family.

Majority 72 (80.9%) father of female students were literate. Among them, almost half 34 (47.2%) have attained secondary level of education. Almost three fourth 66 (74.2%) mother of female students were literate. Among them, 29 (43.9%) have completed secondary level of education.

Cent percent of the students have heard about violence. More than two third 64 (71.9%) have answered the act that is perpetrated against someone's will followed by abusive sexual act 49 (55.1%). Majority 82 (92.1%) of the students had answered female as the most vulnerable sex for violence (Table 1).

**Table 1.** Knowledge regarding meaning of sexual violence.

Variables	n (%)
Heard about sexual violence	
Yes	89 (100)
If yes, meaning of sexual violence*	
Penetrated Vaginal or Anal Sex against someone's will	64 (71.9)
Abusive sexual act (i.e., unwanted touching)	49 (55.1)
Non contact sexual abuse	44 (49.4)
Completed nonconsensual sex act	34 (38.2)
Most vulnerable Sex	
Male	7 (7.9)
Female	82 (92.1)

* *Multiple response questions*

**Figure 1.**
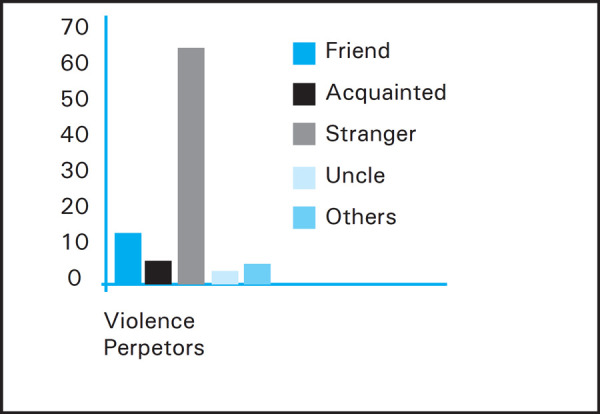
Violence Perpetors.

More than three fourth 71 (79.8%) of the students were victim of violence. Majority 63 (70.8%) of them were victimized from stranger followed by friend 11 (12.4%) (Figure 1).

**Figure 2.**
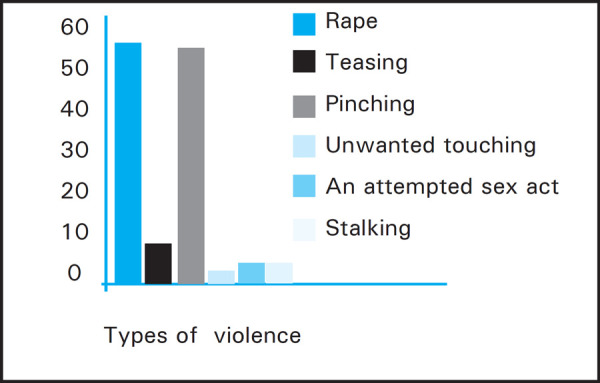
Types of Violence.

Teasing was the most common type of violence 55 (61.8%) followed by unwanted touching 35 (39.3%) (Figure 2).

**Figure 3.**
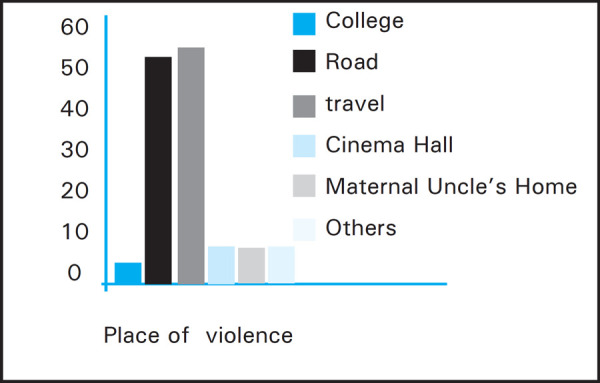
Place of Violence.

Most 51 (57.3%) were the victim while traveling by public vehicle followed by walking on road 47 (52.8%) (Figure 3).

More than three fourth of them 67 (75.3%) were victimized during 11-19 years old. More than half 41 (57.7%) were victimized less than five times and during summer season respectively. Majority 12 (81.7%) of the girls were victimized during evening time. Most of the offenders 19 (43.6%) belong to young adult. Majority 66 (93%) of the offenders had not consumed alcohol or any substances.

More than three fourth 71 (79.8%) of the students would like to inform to the concerned authority after being victimized. Only 18 (20.2%) of them told they would not like to inform because of various reasons like police neglect such issues 4 (22.2%), just want to neglect 3 (16.7%), don't want to disclose 3 (16.7%), no courage to say 3 (16.7%) (Table 2).

**Table 2.** Action after being victimized.

Variable	n (%)
Would you like to inform after being victimized	
Yes	71 (79.8)
No	18 (20.2)
If no, why	
Just want to neglect	3 (16.7)
Male dominated society	2 (11.1)
Don't want to disclose	3 (16.7)
No courage	3 (16.7)
Police neglect such issue	4 (22.2)
No action is taken against them	1 (5.6)
All show mistake to girls	1 (5.6)
Affect family's reputation	1 (5.6)

Regarding the views of the students for the prevention of sexual violence, more than one third 34 (38.2%) told strict punishment to the offenders followed by awareness programme 32 (36%), strict cyber violence laws 22 (24.7%), strict rules and regulations 17 (19.1%), capital punishment to the offenders 16 (18%) (Table 3).

**Table 3.** Prevention of Violence.

What should be done to prevent violence	n (%)
Capital punishment	16 (18)
Awareness programme	32 (36)
Strict cyber violence laws	22 (24.7)
Women's education	10 (11.2)
Strict rules and regulations	17 (19.1)
Avoidance of crowd in public place	3 (3.4)
Women friendly environment	12 (13.5)
Strict punishment to offenders	34 (38.2)
Presence of security in public places	11 (12.4)
Offender's must be humiliated	5 (5.6)
CCTV monitoring in public places	8 (9.0)
Lifetime imprisonment	11 (12.4)
Equality for male and female	7 (7.9)
Offenders should be hanged	5 (5.6)
Raise voice of women	6 (6.7)
Separate place for boys and girls in public	3 (3.4)
Drug control	1 (1.1)
Agencies should be established	2 (2.2)

## DISCUSSION

One of the studies showed that the lower the income and years of formal education, the greater the rates of violence.^7^ Results in this study also indicated that more than three fourth of the female students (75.3%) were victimized during 11-19 years. That indicates that lower the years of formal education and age more risks of violence.

In this study more than half (57.7%) were victimized during summer season. Similar result was shown by another study where the same percentage of the occurrence rate of abuse during summer season (54%).^4^ This study showed that more than three fourth of the female students' (75.3%) were victimized or had some form of violence. One of the study had showed that more than half the women (51.9%) reported having experienced some form of violence in their lifetime.^8^ That evidence proves that female are more vulnerable than others.

This study showed that stranger and friends were the most common perpetrators of violence. Similar finding was reported by Puri & Cleland (2007) where perpetrators included co-workers, boyfriends, employers, and relatives.^6^ A study data showed that: 21% of college students reported having experienced dating violence by a current partner and 32% experienced dating violence by a pervious partner.^9^ Majority (92.1%) of the students had answered female as the most vulnerable sex for violence which is supported by WHO multidisciplinary study which showed women consistently reported higher rates of violence than men.^8^

Regarding the views of the students for the prevention of violence, more than one third (38.2%) told strict punishment to the offenders followed by awareness programme (36%), strict cyber violence laws (24.7%), strict rules and regulations (19.1%), capital punishment to the offenders (18%) which is supported by the study of Krebs et al., (2007).^4^ One of the study stated that specially campus safety initiatives and bystander education models should be discussed which is supported also this study by encouraging the preventive measures as well as focusing on bystander education models. ^2^ The limitation of the study is that this was conducted only in one institute among limited students.

## CONCLUSIONS

College going female students are more vulnerable to physical, verbal, emotional as well as sexual violence or abuse. Awareness program at various levels has to be conducted to the girls regarding the violence. Strict rules and regulations along with strict punishment to the offenders have to be implemented to reduce the violence among girls. In the long run, this will help to reduce the complications that arise out of violence.

## References

[1] Schraiber LB, d'Oliveira AF, França-Junior I, Diniz S, Portella AP, Ludermir AB, Valença O, Couto MT. (2007). Prevalência da violência contra a mulher por parceiro intimo em regiöes do Brasil. Revista de Saude Publica..

[2] Branch KA, Richards TN, Dretsch EC. (2013). An exploratory analysis of college students' response and reporting behavior regarding intimate partner violence victimization and perpetration among their friends. J Interpers Violence..

[3] Csorba R, Tsikouras P, Lampé R, Pöka R. (2012). The sexual abuse of female children in Hungary: 20 years' experience. Archives of gynecology and obstetrics.

[4] Krebs CP, Lindquist CH, Warner TD, Fisher BS, Martin SL. (2009). The differential risk factors of physically forced and alcohol-or other drug-enabled sexual assault among university women. Violence and Victims.

[5] Godia PM, Olenja JM, Hofman JJ, Van Den Broek N. (2014). Young people's perception of sexual and reproductive health services in Kenya. BMC health services research..

[6] Lamichhane P, Puri M, Tamang J, Dulal B. (2011). Women's status and violence against young married women in rural Nepal. BMC women's health.

[7] Krebs CP, Lindquist CH, Warner TD, Fisher BS, Martin SL. (2007). The campus sexual assault (CSA) study: Final report. Washington, DC: National Institute of Justice, US Department of Justice.

[8] Han A, Stewart DE. (2014). Maternal and fetal outcomes of intimate partner violence associated with pregnancy in the Latin American and Caribbean region. Int J Gynecol Obstet..

[9] Tjaden PG, Thoennes N. Extent, nature, and consequences of rape victimization: Findings from the National Violence Against Women Survey.

